# The association of infrared imaging findings of the breast with prognosis in breast cancer patients: an observational cohort study

**DOI:** 10.1186/s12885-016-2602-9

**Published:** 2016-07-27

**Authors:** Li-An Wu, Wen-Hung Kuo, Chin-Yu Chen, Yuh-Show Tsai, Jane Wang

**Affiliations:** 1 Department of Medical Imaging, Taipei City Hospital, Heping Branch, 33, Sec 2, Zhonghua Road, Zhongzheng Dist, Taipei 100, Taiwan; 2 Department of Medical Imaging, National Taiwan University Hospital, 7 Chung-Shan South Road, Taipei 100, Taiwan; 3 Department of Radiology, National Taiwan University College of Medicine, 1, section 1, Jen-Ai Road, Taipei 100, Taiwan; 4 Department of Surgery, National Taiwan University Hospital and National Taiwan University College of Medicine, Taipei, Taiwan; 5 Department of Radiology, Chi-Mei Medical Center, 901 Zhonghua Road, Yongkang District, Tainan 710, Taiwan; 6 Department of Biomedical Engineering, Chung Yuan Christian University, 200 Chung Pei Road, Chung Li Dist, Taoyuan, 32023 Taiwan; 7 Department of Radiology, Taipei Veterans General Hospital, 201, Section 2, Shipai Road, Taipei 112, Taiwan

**Keywords:** Infrared imaging, Breast carcinoma, Prognosis, Mortality

## Abstract

**Background:**

To evaluate whether infrared (IR) imaging findings are associated with prognosis in patients with invasive breast carcinomas.

**Methods:**

This study was approved by the institutional review board of the research ethics committee of our hospital, and all participants gave written informed consent. From March 2005 to June 2007, we enrolled 143 patients with invasive breast cancer that underwent preoperative IR imaging. We used five IR signs to interpret breast IR imaging. Cox proportional hazards model was used to evaluate the effect of IR signs on long-term mortality.

**Results:**

During a median follow-up of 2451 days (6.7 years), 31 patients died. Based on the Cox Proportional Hazards Model, IR1 sign (the temperature of cancer site minus that of the contralateral mirror imaging site) was positively associated with mortality in the univariate analysis (overall mortality hazard ratio [HR], 2.29; *p* = 0.03; disease-specific mortality HR, 2.57; *p* = 0.04) as well as the multivariate analysis after controlling for clinicopathological factors (overall mortality HR, 3.85; *p* = 0.01; disease-specific mortality HR, 3.91, *p* = 0.02). In patients with clinical stage I and II disease, IR1 was also positively associated with mortality (overall mortality HR, 3.76; *p* = 0.03; disease-specific mortality HR, 4.59; *p* = 0.03). Among patients with node-negative disease, IR1 and IR5 (asymmetrical thermographic pattern) were associated with mortality (*p* = 0.04 for both IR1 and IR5, chi-squared test).

**Conclusion:**

Breast IR findings are associated with mortality in patients with invasive breast carcinomas. The association remained in patients with node-negative disease.

**Trial registration:**

NCT00166998.

## Background

Infrared (IR) imaging of the breast, or breast thermography, is a noninvasive modality that measures the surface temperature of the breasts [1–3]. The localized blood flow and metabolic activity of breast cancer are higher than those in normal breast tissue, therefore, the surface temperature overlying the breast cancer is increased [1–3]. Nonetheless, IR imaging has been disregarded in the past due to several concerns including a lack of a standardized protocol, technical difficulties, subjective interpretation, suboptimal sensitivity and specificity for lesion diagnosis, and no direct aid for spatial localization for surgical removal of a tumor [2–7]. However, several studies found that IR imaging was a valuable modality for predicting the risk of breast cancer development and survival [8–20]. In addition, abnormal thermography was associated with advanced tumor staging and metastasis to the lymph nodes [17, 19]. Recently, digital breast IR imaging has resurfaced as an adjunct to mammography in diagnosing breast cancer, especially in dense breast tissue [21, 22]. Moreover, some integrated interpretive models using several different IR signs were also developed [2, 21, 23]. Digital IR was also reported to be associated with prognosis in breast cancer patients [2, 22, 24–27]. Ohsumi et al. [22] reported that increased mean temperature (ΔT > = 0.9 °C) of the tumor area in comparison to that of the corresponding area of the contralateral breast was an independent significant prognostic factor for disease-specific survival. However, they also reported that ΔT did not have any prognostic impact on the patients with node-negative disease [22]. Wang et al. [23] reported that breast IR signs were related to molecular subtypes, clinical staging and histologic grade of breast cancer, however, the analysis of survival was not performed in this study.

In our current study, we evaluated the prognostic value of breast IR imaging by five IR signs (Table 1). In addition, we also evaluated the prognostic value of IR imaging signs in patients with node-negative disease and patients with stage I or II breast cancer, which was previously reported to be statistically insignificant [22].

**Table 1:** Descriptions of infrared (IR) imaging signs

Parameter	Description of sign
IR1	Temperature difference (ΔT) of the lesion site from the mirror image site of the contralateral breast. IR1 = 0 (negative) when ΔT ≤ 2 °C; IR1 = 1 (positive) when ΔT > 2 °C.
IR2	Temperature difference of the lesion site from the adjacent normal breast. IR2 = 0 (negative) when ΔT ≤ 1 °C; IR2 = 1 (positive) when ΔT > 1 °C.
IR3	Abnormal vascular morphologic patterns at and around the tumor. IR3 = 0 when the sign is absent; IR3 = 1 when the sign is present.
IR4	Focal edge or bulge of the surface contour with increased temperature. IR4 = 0 when the sign is absent; IR4 = 1 when the sign is present.
IR5	Asymmetric thermographic and vascular patterns at the tumor site. IR5 = 0 when the sign is absent; IR5 = 1 when the sign is present.

The table content was reprinted with permission and adapted from Wang et al., BioMedical Engineering OnLine 2010; 9:3. Doi:10.1186/1475-925X-9-3 (Publisher: BioMed Central Ltd, part of Springer Science + Business Medica) [2], and Wang et al., Academic Radiology 2011; 18(2): 212–219 (Publisher: Elsevier) [23]

## Methods

### Patient enrollment

From March 2005 to June 2007, we enrolled 143 patients with pathologically proven invasive breast carcinoma, and all of them underwent breast IR imaging before operation. Those who received neoadjuvant chemotherapy (NAC) before operation were excluded from our study because the breast IR was performed only at pre-treatment stage, and the disease status after NAC would change and cannot be comparable with that of pre-treatment IR. The study participants were a subgroup of the patients in our previous studies [2, 23], one of which investigated the diagnostic performance of different IR signs (298 lesions from 276 women, including 174 breast cancer lesions from 165 patients) [2], and the other prior study dealt with the assessment of the association of IR signs with molecular subtypes of breast cancer, including Estrogen Receptor, Progesterone Receptor, and Human Epidermal Growth Factor Receptor 2 in 171 breast cancer lesions from 163 patients [23]. In our current study, we report on the role of IR signs in predicting the prognosis in women with invasive breast carcinomas. This study was approved by the institutional review board of the research ethics committee of our hospital, and all participants gave written informed consent before the IR examination.

### IR imaging protocol and interpretation

The IR procedure, image processing, and interpretation were conducted as previously described [2, 23]. The IR imaging of the breast was performed using the ATIR-M301 Thermal Imaging System (response wavelength of 8 to 12 mm; Associated Technology Corporation, Chongqing, Sichuan, China). The examination room was maintained at a constant temperature of 23 °C to 25 °C. Each participant removed their upper outer garment and then sat on a chair for 15 min, and the IR images were then taken. The post-processing of IR images was performed using M301-APP-V2.0 software (Associated Technology Corporation). The location and size of the lesions were marked by a radiologist (first radiologist) based on the mammography and ultrasound studies and were then recorded on a sheet. The other two radiologists (second and third radiologists) interpreted the IR images, and their interpretation was based only on the information from the previously recorded sheet. The two IR imaging readers were blinded to the detailed mammographic and ultrasonographic findings, pathologic results of the patients, and the two radiologists were both specialized in breast imaging for more than 10 years.

The five IR signs we used in this study (Table 1) were modified from those described in the literature [2, 21, 23]. Besides, because of insignificant prognostic impact of ΔT in node-negative patients as previously reported (ΔT > =0.9 °C temperature difference of both breasts) [22], we adjusted the positive IR1 sign as >2 °C difference in the temperature (ΔT) of the lesion site from that of the contralateral breast.

### Survival analysis

All data were analyzed using IBM SPSS Statistics software, version 21 (IBM SPSS Statistics for Windows, Version 21.0. Armonk, NY: IBM Corp.). The primary outcomes were overall mortality and disease-specific mortality. The follow-up period was defined as starting at the diagnosis of breast cancer and ending on the date of death, the date they were last known to be living, or the date of the most recent follow-up. The last date of data collection was December 31, 2014, and patients for whom no event had occurred or who were lost to follow-up were censored accordingly. The clinical follow-up and survival data of all patients were retrieved from Cancer Registry, Cancer Administration and Coordination Center of our hospital.

The univariate Cox proportional hazards model was used to analyze the association of the five IR signs (IR1 to IR5) and the clinicopathological variables (age, menopausal status, clinical stage, histologic type, nuclear grading, and molecular subtypes) with overall mortality and disease-specific mortality. A multivariate Cox proportional hazards model was also used to analyze the correlation of the IR signs with overall mortality and disease-specific mortality after adjusting for the clinicopathological variables. The association between the IR signs and overall survival in patients with node-negative and node-positive disease was evaluated using the chi-squared test. *P* values < 0.05 were considered to indicate statistical significance.

The overall and disease-specific survivals were estimated using the Kaplan-Meier method. The log-rank test was used to compare the survival curves between groups categorized by different IR signs.

## Results

In total, 143 patients with primary invasive breast cancer were enrolled in this study (age range, 27–81 years; mean age, 54.2 years). Table 2 summarizes the clinical data of the patients. Of them, 101 (70.6 %) patients had stage I and II disease, and just over half of the patients (78/143, 54.5 %) had the luminal molecular subtype. As for the histologic type, 130 patients (90.9 %) had invasive ductal carcinomas, eight patients (5.6 %) had invasive lobular carcinomas, and five patients (3.5 %) had other histologic types of carcinoma. Of the 143 patients, 136 (95 %) had unilateral breast disease and seven of them (5 %) had bilateral synchronous breast cancers. All the patients underwent definitive surgery followed by appropriate adjuvant therapy including chemotherapy, hormone therapy, or targeted therapy, radiotherapy according to National Comprehensive Cancer Network (NCCN®) guidelines (Fort Washington, PA, USA) for breast cancer.

**Table 2:** Clinical data of the 143 patients with breast cancer

Variable	N (%)
Menopausal status	
Premenopausal	45 (31.5)
Postmenopausal	98 (68.5)
Bilateral breast cancer	7 (5)
Clinical stage	
Stage I and II	101 (70.6)
Stage III	31 (21.7)
Stage IV	11 (7.7)
Molecular subtype	
ER/PR-positive, HER2-negative	78 (54.5)
HER2-positive	32 (22.4)
Triple negative	33 (23.1)
Histology types	
Invasive ductal carcinoma	130 (90.9)
Grade 1	15 (10.5)
Grade 2	68 (47.5)
Grade 3	39 (27.3)
Unknown	8 (5.6)
Other cancer types	
Invasive lobular carcinoma	8 (5.6)
Apocrine carcinoma	1 (0.7)
Mucinous carcinoma	1 (0.7)
Intracystic papillary carcinoma	2 (1.4)
Metaplastic carcinoma	1 (0.7)

*ER* estrogen receptor, *PR* progesterone receptor, *HER2* human epidermal growth factor receptor 2

The median follow-up period for all of the patients was 2451 days (6.7 years; range, 172–2920 days). During this period, 31 (22 %) patients died. Among the deceased patients, 28 (90.3 %) died of breast cancer, and three died due to other unrelated disorders (one of pancreatitis, another of liver cirrhosis, and the etiology of the death of the remaining patients was undetermined).

### Survival analysis

For the univariate analysis, a high clinical stage was associated with poor survival (overall mortality hazard ratio [HR]: 1 [stages I and II], 3.00 [stage III], 10.89 [stage IV], *p* < 0.0001; disease-specific mortality HR: 1 [stages I and II], 1.92 [stage III], 14.02 [stage IV]; *p* < 0.0001). Age, menopausal status, histological grade, pathologic type, and molecular subtype were not significantly associated with mortality. The univariate analysis of the IR signs revealed that a positive IR1 (ΔT > 2 °C) was associated with poor survival (overall mortality HR, 2.29; *p* = 0.03; disease-specific mortality HR, 2.57; *p* = 0.04). Other IR factors were not significantly associated with mortality in univariate analysis (Table 3).

**Table 3:** Univariate analysis of overall mortality and disease-specific mortality

		Overall mortality			Disease-specific mortality		
Variable	n	HR	95 % CI	*p*	*HR*	95 % CI	*p*
Age ≤ 50 years	51	1		0.92	1		0.45
Age > 50 years	92	0.96	0.44–2.08		1.49	0.54–4.12	
Postmenopausal	85	1.06	0.50–2.27	0.87	0.94	0.38–2.34	0.89
Clinical stage				<0 .0001			<0.0001
stages I & II	101	1			1		
Stage III	31	3.00	1.24–7.24		1.92	0.58–6.39	
Stage IV	11	10.89	4.35–27.23		14.02	5.05–38.94	
Histology				0.14			0.92
IDC	130	1			1		
ILC	8	2.94	1.02–8.51		1.06	0.14–7.99	
Other subtypes	5	1.07	0.14–7.95		1.51	0.20–11.35	
IDC tumor grade				0.80			0.86
Grade 1	15	1			1		
Grade 2	68	1.84	0.23–14.71		0.58	0.32–1.04	
Grade 3	39	2.10	0.25–17.96		0.40	0.21–0.79	
Molecular subtype				0.39			0.36
Luminal	78	1			1		
HER2-enriched	32	1.73	0.71–4.24		0.87	0.23–3.22	
Triple-negative	33	1.65	0.67–4.03		1.90	0.71–5.11	
IR signs ^a^							
IR1				0.03			0.04
IR1 = 0	109	1			1		
IR1 = 1	34	2.29	1.07–4.89		2.57	1.03–6.40	
IR2				0.75			0.42
IR2 = 0	61	1			1		
IR2 = 1	82	0.89	0.42–1.87		0.69	0.28–1.70	
IR 3				0.33			0.53
IR3 = 0	12	1			1		
IR3 = 1	131	2.71	0.37–19.95		1.91	0.26–14.34	
IR 4				0.50			0.22
IR4 = 0	114	1			1		
IR4 = 1	29	1.34	0.57–3.16		1.83	0.70–4.81	
IR 5				0.47			0.93
IR5 = 0	26	1			1		
IR5 = 1	117	1.48	0.51–4.27		0.95	0.32–2.87	

Estimated by univariate Cox proportional hazards analysis

*HR* hazard ratio, *95 % CI* 95 % confidence interval, *IDC* invasive ductal carcinoma, *ILC* invasive lobular carcinoma; IR: infrared

^a^IR imaging signs are defined in Table 1; 0 = negative, 1 = positive

In the multivariate analysis of IR signs, controlling for clinicopathological factors (including age, clinical tumor stage, pathological tumor grade, molecular subtype), a positive IR1 sign was associated with poor survival outcome (overall mortality HR, 3.85; *p* = 0.01; disease-specific mortality HR, 3.91; *p* = 0.02). The other IR signs were not significantly related to survival outcome (Table 4).

**Table 4:** Association of IR signs and overall mortality and disease-specific mortality after controlling for clinicopathological variables

		Overall mortality			Disease-specific mortality		
Variable	n	HR	95 % CI	*p*	HR	95 % CI	*p*
IR parameter^a^							
IR1				0.01			0.02
IR1 = 0	109	1			1		
IR1 = 1	34	3.85	1.37–10.81		3.91	1.22–12.59	
IR2				0.65			0.30
IR2 = 0	61	1			1		
IR2 = 1	82	0.80	0.30–2.15		0.56	0.18–1.68	
IR3				0.88			0.93
IR3 = 0	12	1			1		
IR3 = 1	131	0.85	0.10–6.96		1.11	0.13–9.44	
IR4				0.98			0.16
IR4 = 0	114	1			1		
IR4 = 1	29	1.01	0.33–3.10		2.26	0.74–6.93	
IR5				0.85			0.60
IR5 = 0	26	1			1		
IR5 = 1	117	1.14	0.31–4.17		0.69	0.18–2.66	

Estimated by multivariate Cox proportional hazards model

Clinicopathological variables: age, clinical tumor staging, pathological tumor grade, molecular subtypes

*HR* hazard ratio, *95 % CI* 95 % confidence interval

^a^IR imaging signs are defined in Table 1; 0 = negative, 1 = positive

Among the 101 patients with stage I or II breast cancer, a positive IR1 sign was also associated with poor survival (overall mortality HR, 3.76; *p* = 0.03; disease-specific mortality HR, 4.59; *p* = 0.03). Other IR factors were not significantly associated with survival outcome (Table 5). Among patients with advanced stage breast cancer (clinical stage III or IV, *n* = 42), no IR sign was significantly associated with overall or disease-specific survival outcomes.

**Table 5:** Association of IR findings with mortality in patients with clinical stage I and II tumors

Variable		Overall mortality			Disease-specific mortality		
	n	HR	95 % CI	*p*	HR	95 % CI	*p*
IR signs^a^							
IR1				0.03			0.03
IR1 = 0	81	1			1		
IR1 = 1	20	3.76	1.15–12.31		4.59	1.15–18.37	
IR2				0.93			0.62
IR2 = 0	46	1			1		
IR2 = 1	55	1.06	0.32–3.46		1.43	0.34–6.00	
IR3				0.45			0.93
IR3 = 0	10	1			1		
IR3 = 1	91	0.68	0.45–1.68		0.92	0.11–7.45	
IR4				0.94			0.22
IR4 = 0	83	1			1		
IR4 = 1	18	0.95	0.20–4.39		2.45	0.58–10.25	
IR5				0.72			0.53
IR5 = 0	19	1			1		
IR5 = 1	82	0.91	0.55–1.51		1.96	0.24–15.95	

Total number of patients with clinical stage I and II tumors = 101

Estimated by Cox proportional hazards model

*HR* hazard ratio, *95 % CI* 95 % confidence interval

^a^IR imaging signs are defined in Table 1; 0 = negative, 1 = positive

There were 69 node-positive and 74 node-negative patients. Among the 74 patients with node-negative disease, the positive IR1 (ΔT > 2 °C) and IR5 signs (asymmetric thermographic pattern) were associated with high overall mortality (*p* = 0.04 for both IR1 and IR5, chi-squared test). The other IR signs showed no significant relation to mortality (Table 6). On the other hand, among node-positive patients (*n* = 69), there was no significant association of IR signs and overall mortality (IR1, IR2, IR3, IR4, and IR5, *p* = 0.77, 0.08, 0.74, 0.41, and 0.05, respectively, chi-square test).

**Table 6:** Prognostic significance of infrared (IR) imaging parameters in patients with node-negative breast cancer

	Deceased	Survived	
IR signs^a^	n (%)	n (%)	*p*
IR1			0.04
IR1 = 0	7	52	
IR1 = 1	5	10	
IR2			0.60
IR2 = 0	5	31	
IR2 = 1	7	31	
IR3			0.26
IR3 = 0	0	6	
IR3 = 1	12	56	
IR4			0.96
IR4 = 0	10	52	
IR4 = 1	2	10	
IR5			0.04
IR5 = 0	0	17	
IR5 = 1	12	45	

Total number of patients with node-negative breast cancer = 74

The number of deceased patients = 12

Estimated by chi-square test

^a^IR imaging signs are defined in Table 1

**Fig. 1:**
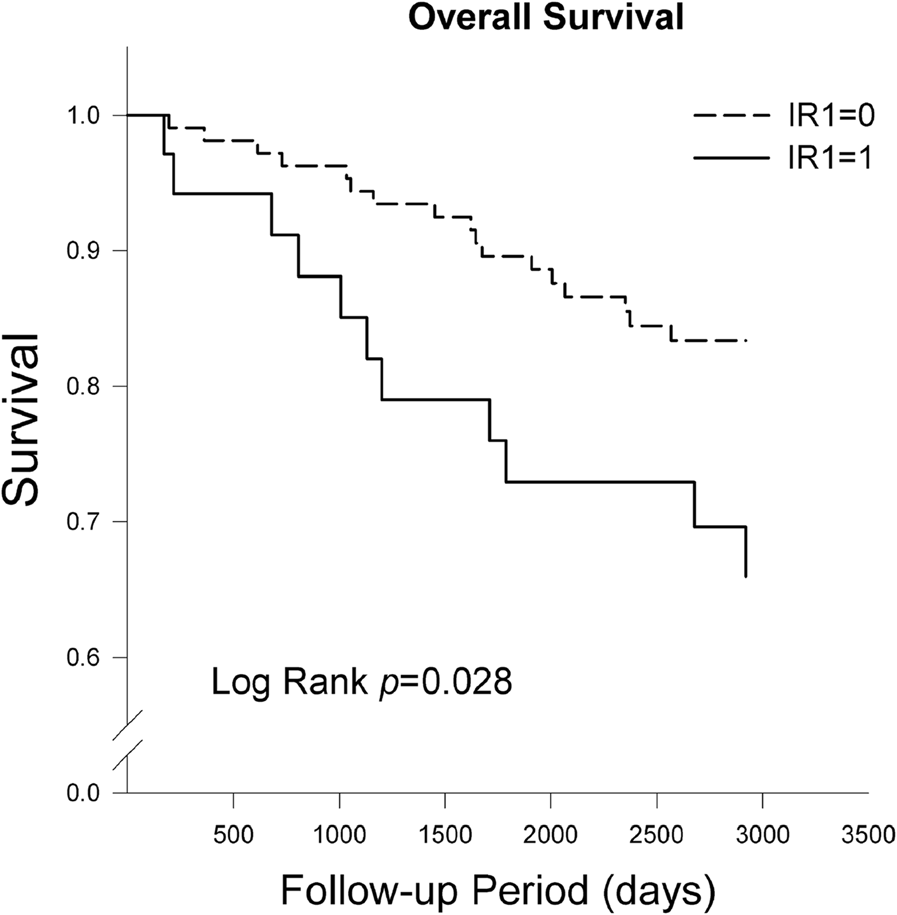
Overall survival by IR1 sign in the Kaplan-Meier analysis. The patients with a positive IR1 sign had significantly poorer overall survival than patients with a negative IR1 sign (*p* = 0.028, log-rank test)

**Fig. 2:**
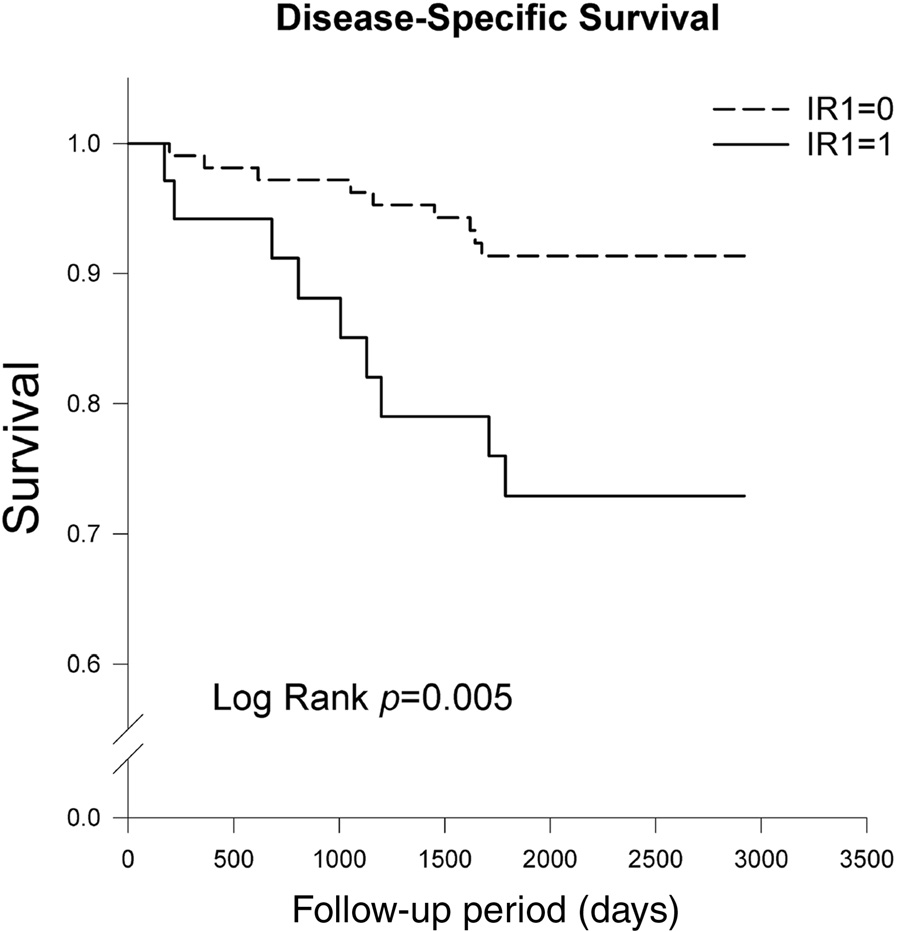
Disease-specific survival by IR1 sign in the Kaplan-Meier analysis. The patients with a positive IR1 sign had significantly poorer disease-specific survival than patients with a negative IR1 sign (*p* = 0.005, log-rank test)

Kaplan-Meier analysis showed that the IR1-positive patients had poorer overall and disease-specific survival rates than the IR1-negative patients (log-rank test, *p* = 0.028 and 0.005 for overall and disease-specific survival, respectively) (Figs. 1 and 2).

## Discussion

We evaluated the association of breast IR signs with survival outcomes. We found that a positive IR1 sign was significantly associated with higher mortality. In addition, after adjusting for clinicopathological variables, the IR1 sign was still a significant independent prognostic factor.

We found that a positive IR1 sign was related to higher overall and disease- specific mortality in patients with stage I and II cancers, and a positive IR1 sign was associated with higher overall mortality in node-negative patients. These findings differed from those of Ohsumi et al. [22], since they found that an abnormal thermogram did not have a prognostic impact on node-negative patients [22]. The different results may be related to the reasons below: first, the cutoff values of ΔT between the two studies were different, that is, ΔT > 2 °C (for IR1 sign) in our study versus ΔT > = 0.9 °C in their study; second, we used a staging system incorporating tumor size (T), nodal status (N) and metastasis (M) as one of the clinicopathological variables, while the Ohsumi’s study analyzed tumor size, nodal status separately.

IR signs were not associated with survival outcomes in women with stage III or IV breast cancer or in node-positive patients in our study. The prognosis of these patients is mainly related to the status of lymph node metastasis or systemic metastasis, which may not have been well evaluated in IR imaging since small lymph nodes metastasis or visceral organ metastasis may not show detectable surface temperature changes. In addition, the abnormal lymph nodes deeper to pectoral muscles are hard to be detected by IR. Although several previous studies stated that patients with abnormal thermograms had significantly larger tumors and higher percentage of metastatic lymph nodes than patients with normal thermograms, there was still no proof of the prognostic value of thermography in the patients with advanced breast cancer [17, 19, 22].

On the other hand, we found an asymmetric thermographic pattern (positive IR5 sign) was related to the higher overall mortality in the patients without lymph node metastasis. This was not surprising since an asymmetric thermographic pattern is a morphologically descriptive sign that may reflect asymmetric surface temperatures [2], and thus IR5 has a similar implication to IR1 sign. Therefore, breast IR signs can be potential imaging markers to predict prognosis in selected subgroups of breast cancer patients, that is, patients with stage I, II cancers or node-negative patients as stated above.

There are some limitations in this study. The sample size was limited; therefore, we did not further stratify the molecular subtypes in more detail. In addition, the treatment protocols were heterogeneous due to different molecular subtypes and stages, which may influence survival. We did not include patients that underwent neoadjuvant chemotherapy and we were not able to perform serial breast IR studies to monitor treatment response of neoadjuvant chemotherapy. Finally, we did not compare the IR imaging to other diagnostic modalities, such as mammography, ultrasound, MRI, or PET.

## Conclusions

IR1 imaging sign could be a potential imaging marker to predict prognosis in patients with invasive breast cancers with stage I, II or node-negative disease. IR5 sign was associated with overall mortality in breast cancer patients with node-negative disease. In the future, recruitment of more study participants to validate our results, and use of IR to monitor treatment response for patients with neoadjuvant chemotherapy are needed.

## Abbreviations

CI, confidence interval; ER, estrogen receptor; HER2, human epidermal growth factor receptor 2; HR, hazard ratio; IDC, invasive ductal carcinoma; ILC, invasive lobular carcinoma; IR imaging, Infrared imaging; PET, positron emission tomography; PR, progesterone receptor
